# 100 top-cited scientific papers in limb prosthetics

**DOI:** 10.1186/1475-925X-12-119

**Published:** 2013-11-17

**Authors:** Arezoo Eshraghi, Noor Azuan Abu Osman, Hossein Gholizadeh, Sadeeq Ali, Babak Shadgan

**Affiliations:** 1 Department of Biomedical Engineering, Faculty of Engineering, University of Malaya, 50603 Kuala Lumpur, Malaysia; 2 Center for International Collaboration On Repair Discoveries, University of British Columbia, Vancouver, Canada

**Keywords:** Citation-classics, Orthotics, Prosthetics, Journals, Association, Archives, Science, Bias

## Abstract

Research has tremendously contributed to the developments in both practical and fundamental aspects of limb prosthetics. These advancements are reflected in scientific articles, particularly in the most cited papers. This article aimed to identify the 100 top-cited articles in the field of limb prosthetics and to investigate their main characteristics. Articles related to the field of limb prosthetics and published in the Web of Knowledge database of the Institute for Scientific Information (ISI) from the period of 1980 to 2012. The 100 most cited articles in limb prosthetics were selected based on the citation index report. All types of articles except for proceedings and letters were included in the study. The study design and level of evidence were determined using Sackett’s initial rules of evidence. The level of evidence was categorized either as a systematic review or meta-analysis, randomized controlled trial, cohort study, case–control study, case series, expert opinion, or design and development. The top cited articles in prosthetics were published from 1980 to 2012 with a citation range of 11 to 90 times since publication. The mean citation rate was 24.43 (SD 16.7) times. Eighty-four percent of the articles were original publications and were most commonly prospective (76%) and case series studies (67%) that used human subjects (96%) providing level 4 evidence. Among the various fields, rehabilitation (47%), orthopedics (29%), and sport sciences (28%) were the most common fields of study. The study established that studies conducted in North America and were written in English had the highest citations. Top cited articles primarily dealt with lower limb prosthetics, specifically, on transtibial and transradial prosthetic limbs. Majority of the articles were experimental studies.

## Introduction

Limb prosthetics have a significant function in the field of rehabilitation. The need for prostheses has increased as a result of the increasing rate of diabetes, trauma, and increased life expectancy. Studies in the field of prosthetics have been conducted since World War II. Although practical and clinical skills have constituted the bases for progress in the field of rehabilitation, research has tremendously contributed to the developments in both practical and fundamental aspects. These advancements are reflected in scientific articles, particularly in the most cited papers. Reviewing the top cited articles can provide insights into studies that have contributed the most to these developments. A growing trend is to publish data related to the most cited articles in various clinical disciplines, such as orthopaedics, emergency medicine, plastic surgery, rehabilitation, toxinology, and Parkinson’s disease [1-6].

The Institute for Scientific Information (ISI) initiated the Science Citations Index in 1962 as a means of measuring citation numbers for scientific journals in a systematic and constant manner. Bibliometrics, which refers to the study and analysis of citation indexes, has led to the development of different metrics to evaluate the impact of scientific journals or researchers according to the number of times their works have been cited by other authors [7]. In the present study, we employed these tools to determine the key works in prosthetics limb studies.

A number of studies have reported the top cited articles in the field of rehabilitation. Shadgan et al. identified the 100 most cited articles published in journals within the scope of rehabilitation [4]. Physical therapy is among the rare areas of concern in the rehabilitation field that has undergone citation analysis [8-10]. However, no comprehensive paper has dealt with top cited articles in prosthetics. This review is an attempt to identify the 100 most cited papers related to prosthetics to help clinicians and researchers determine areas of research in prosthetics that have been extensively addressed as well as the gaps that should be addressed.

## Methods

A search for all articles relevant to “prosthetic limb” was conducted through the ISI Web of Knowledge database because this study only aimed to search for articles dealing with prosthetic limbs and relevant issues. Science Citation Index Expanded (SCI-EXPANDED) was adopted as the citation database. The following keywords, single or combined, as well as their synonyms were used to find articles: transfemoral prosthesis, transtibial prosthesis, artificial limb, lower limb prosthesis, leg prosthesis, transradial prosthesis, transhumeral prosthesis, knee disarticulation prosthesis, hand prosthesis, upper limb prosthesis, leg prosthesis, arm prosthesis, artificial arm, artificial leg, limb prosthesis, prosthetic limb. The search results were filtered to exclude papers related to implant prostheses. Proceedings, editorials, and letters were excluded by refinement. The articles published from 1980 to 2012 were retrieved. The final filtered papers were ranked from most to least cited. The first 100 papers were inputted into an Excel spreadsheet.

**Figure 1:**
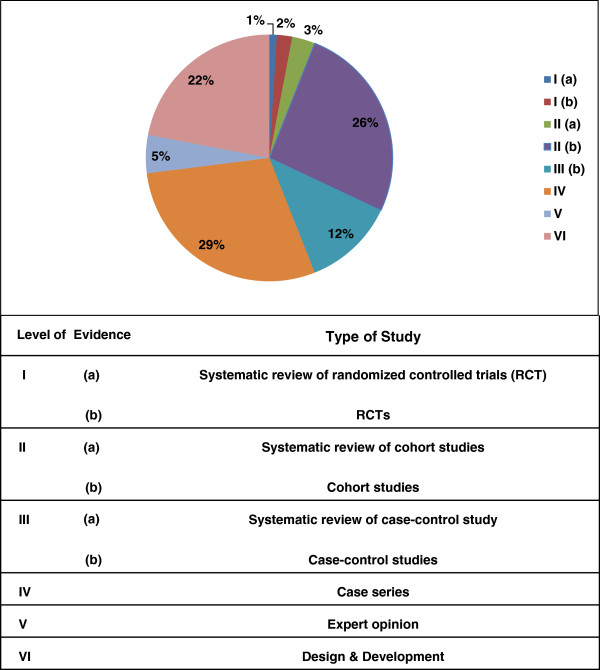
Distribution of the articles with regards to the levels of evidence.

Several articles were cited more often than others because of the difference in time since publication. A citation index was also determined for each article to control this error. The citation index was defined as the mean number of citation times per year [3]. The 100 top cited articles were then identified based on the number of citations and citation index value (Additional file 1: Table S1). The articles were classified based on article specifications, such as publication date, journal name, title, authors’ names, organization, and country. The data collection method was categorized as retrospective, prospective, cross-sectional, and longitudinal. The research design was likewise defined to be qualitative, quantitative or mixed mode [11]. We categorized the papers based on anatomy, such as lower and upper limbs. For both upper and lower limb prostheses, the articles were further categorized based on the type of prosthesis, including transfemoral (TF), transtibial (TT), knee disarticulation (KD), Syme’s, and partial foot for lower limb, and shoulder disarticulation (SD), transradial (TR), transhumeral (TH), wrist disarticulation, and partial hand for upper limb. Sackett’s initial rules of evidence were used to score the articles based on their levels of evidence (Figure 1) [12].

A sixth category called “Design and Development” was added because several articles dealt with the design and development of prosthetic devices without clinical analysis on humans.

Statistical analysis was performed using SPSS 20 (SPSS Inc., Chicago IL). Non-parametric test of Kruskal Wallis was used to compare the differences with an alpha level of .05. Descriptive statistics were quantified as counts or percentages of parameters.

## Results

### Sources and citations

One hundred selected articles were published from 1981 to 2011 with a citation range of 11 to 90 times since publication. The average citation rate was 24.43 (SD 16.7) times. The citation count was used to sort the most cited articles. Among the 100 articles, 16 were from the Journal of Rehabilitation Research and Development, 11 from the Archives of Physical Medicine and Rehabilitation, and 9 from the Prosthetics and Orthotics International. The highest number of articles (11) was published in 2004. The highest number of citation, 90, was related to a paper on upper limb prosthetics that was published in 2004. More articles were published from 2001 to 2011 compared with that in 1991 to 2001 (55 vs. 40). Of all the papers, 47% were published in the Web of Science category of rehabilitation, followed by orthopedics (29%), sport sciences (28%), biomedical engineering (20%), and neurosciences (11%). The rest of the categories covered 10 or less than 10 papers. Tables 1 and 2 present the first 10 top cited articles in the field of lower limb and upper limb prosthetics, respectively.

**Table 1:** The first 10 top cited articles in the field of lower limb prosthetics

**Article**	**No. of citation**
Houghton A, Taylor P, Thurlow S, Rootes E, McColl I. Success rates for rehabilitation of vascular amputees: implications for preoperative assessment and amputation level. Brit J Surg. 1992;79:753–5.	89
Schmalz T, Blumentritt S, Jarasch R. Energy expenditure and biomechanical characteristics of lower limb amputee gait: The influence of prosthetic alignment and different prosthetic components. Gait posture. 2002;16:255–63.	71
McWhinnie D, Gordon A, Collin J, Gray D, Morrison J. Rehabilitation outcome 5 years after 100 lower‒limb amputations. Brit J Surg. 1994;81:1596–9.	67
Legro MW, Reiber G, del Aguila M, Ajax MJ, Boone DA, Larsen JA, et al. Issues of importance reported by persons with lower limb amputations and prostheses. J Rehab Res Dev. 1999;36:155–63.	51
Nolan L, Wit A, Dudziñski K, Lees A, Lake M, Wychowañski M. Adjustments in gait symmetry with walking speed in trans-femoral and trans-tibial amputees. Gait Posture. 2003;17:142–51.	45
Sanderson DJ, Martin PE. Lower extremity kinematic and kinetic adaptations in unilateral below-knee amputees during walking. Gait Posture. 1997;6:126–36.	43
Isakov E, Mizrahi J, Ring H, Susak Z, Hakim N. Standing sway and weight-bearing distribution in people with below-knee amputations. Arch Phys Med Rehabil. 1992;73:174.	43
Hof AL, van Bockel RM, Schoppen T, Postema K. Control of lateral balance in walking: experimental findings in normal subjects and above-knee amputees. Gait Posture. 2007;25:250–8.	39
Pezzin LE, Dillingham TR, MacKenzie EJ, Ephraim P, Rossbach P. Use and satisfaction with prosthetic limb devices and related services. Arch Phys Med Rehabil. 2004;85:723–9.	39
Sup F, Bohara A, Goldfarb M. Design and control of a powered transfemoral prosthesis. Int J Robot Res. 2008;27:263–73.	36

**Table 2:** The first 10 top cited articles in the field of upper limb prosthetics

**Article**	**No. of citation**
Kuiken T, Dumanian G, Lipschutz R, Miller L, Stubblefield K. The use of targeted muscle reinnervation for improved myoelectric prosthesis control in a bilateral shoulder disarticulation amputee. Prosthet Orthot Int. 2004;28:245–53.	90
Jacobson SC, Knutti DF, Johnson RT, Sears HH. Development of the Utah artificial arm. IEEE Trans Biomed Eng. 1982; 29:249–69.	83
Karlik B, Osman Tokhi M, Alci M. A fuzzy clustering neural network architecture for multifunction upper-limb prosthesis. IEEE Trans Biomed Eng. 2003;50:1255–61.	63
Light C, Chappell P. Development of a lightweight and adaptable multiple-axis hand prosthesis. Med Eng Phys. 2000;22:679–84.	53
Pons J, Rocon E, Ceres R, Reynaerts D, Saro B, Levin S, et al. The MANUS-HAND dextrous robotics upper limb prosthesis: mechanical and manipulation aspects. Autonom Robot. 2004;16:143–63.	40
Carrozza M, Massa B, Micera S, Lazzarini R, Zecca M, Dario P. The development of a novel prosthetic hand-ongoing research and preliminary results. IEEE/ASME Tran Mech. 2002;7:108–14.	37
Farrell TR, Weir RF. The optimal controller delay for myoelectric prostheses. IEEE Tran Neur Sys Rehabil Eng. 2007;15:111–8.	36
Ehrsson HH, Rosén B, Stockselius A, Ragnö C, Köhler P, Lundborg G. Upper limb amputees can be induced to experience a rubber hand as their own. Brain. 2008;131:3443–52.	35
Biddiss EA, Chau TT. Upper limb prosthesis use and abandonment: A survey of the last 25 years. Prosthet Orthot Int. 2007;31:236–57.	29
Sebelius FC, Rosen BN, Lundborg GN. Refined myoelectric control in below-elbow amputees using artificial neural networks and a data glove. J Hand Surg. 2005;30:780–9.	27

### Study field and journal distribution

**Figure 2:**
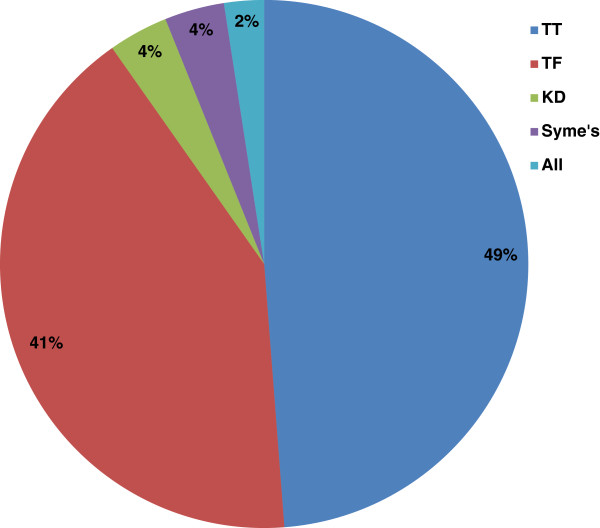
**The percentage of coverage for every prosthesis type in the lower limb prosthetics.** TT = transtibial; TF = transfemoral; KD = knee disarticulation.

**Figure 3:**
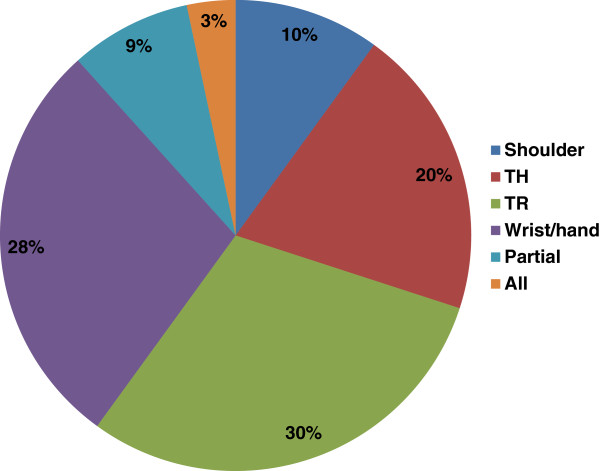
**The percentage of coverage for every prosthesis type in upper limb prosthetics.** TH = transhumeral; TR = transradial.

The articles were classified based on limb amputation of either lower or upper limb. Among the top 100 papers, the greatest number was on lower limb prostheses (53 articles). Only 6% of the articles addressed both lower and upper limb prosthetics. The articles on lower limb dealt with the following amputation levels from the highest to lowest: TT (40 times), TF (34 times), KD (3 times), and Syme’s (3 times). Among the articles on upper limb prostheses, papers that addressed TR level of amputation had the highest rate (18 articles), followed by wrist (17 articles), and TH (12 articles). Only two papers had studied all upper limb amputation levels. Figures 2 and 3 illustrate the percentage of coverage for every prosthesis type in lower and upper limb prosthetics, respectively.

More top cited articles from the United States (the first country with the most articles) focused on the lower limb than the upper limb (18 vs. 13), but no significant difference was observed. This rate was 10 (lower limb) to 6 (upper limb) for the United Kingdom and 8 to 4 for Canada. The United States and United Kingdom had two and three studies, respectively, on both upper and lower limb prostheses in mixed mode.

**Figure 4:**
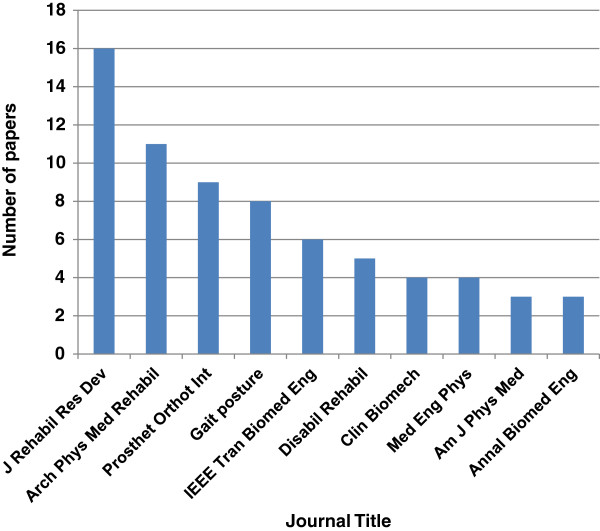
The top 10 journals that published the highest number of top cited papers.

The first five journals with the highest number of most cited articles include: 1) Journal of Rehabilitation Research and Development (17%); 2) Archives of Physical Medicine and Rehabilitation (11%); 3) Prosthetics and Orthotics International (9%); 4) Gait and Posture (8%); and 5) IEEE Transactions on Biomedical Engineering (6%). In total, the 100 top cited papers obtained 2443 citations, from which the highest number of citations was related to the Journal of Rehabilitation Research and Development with 333 times, followed by 205 times for the Prosthetics and Orthotics International, and 199 times for the Archives of Physical Medicine and Rehabilitation. A significant difference was observed between journals in terms of citation (*P* = .016). Although in terms of ranking, Prosthetics and Orthotics International had a fewer number of top cited articles than Archives of Physical Medicine and Rehabilitation, it gained higher number of citations. Figure 4 illustrates the top 10 journals that published the highest number of top-cited papers.

### Type of study and level of evidence

Studies included 2 RCT, 26 cohort, 12 case–control, 21 case series, and 8 case studies. Twenty-two articles were categorized under the “Design and Development” (level 6) or used finite element methods. Among the articles on the design and development of prosthetics, majority were on upper limb prostheses (16 papers), whereas only one article dealt with lower limb prosthetics. One paper was related to both upper and lower limb amputation levels (3D imaging of residual limbs by using ultrasound).

Majority (69 articles) of the top 100 most cited articles were original studies conducted on human subjects. Four review papers, from which two were on the lower limb prostheses and two on the upper limb, were found. Sixty percent of the studies adopted quantitative research design compared with only 39% that adopted the qualitative design. Only 2.9% of the studies involved longitudinal data collection. Most studies adopted the cross-sectional method of data collection.

Figure 1 illustrates the distribution of articles with regard to the level of evidence.

### Authors, organizations and countries of origin

The top cited papers were written by 308 authors. The mean number of authors per article was 3.76 (SD 1.81). Four articles had a single author, whereas majority of the articles had eithertwo (n = 25) or three authors (n = 26). Out of 100 papers, 31 had five or more authors. The maximum number of authors was nine (one article). Several authors contributed to more than one paper. The most frequent first author of the top cited articles was W.C.C Lee (n = 3). Goldfarb and Kuiken each contributed to five papers (Table 3). Kuiken was the first author in only one article; Goldfarb had no articles published as the first author.

**Table 3:** Authors of the most cited articles in prosthetics

**Author**	**Total articles**	**As first author**
Goldfarb M	5	0
Kuiken TA	5	1
Czerniecki JM	4	1
Lundborg G	4	2
Rosen B	4	1
Chappell PH	3	1
Gitter A	3	1
Lee WCC	3	3
Miller LA	3	2
Postema K	3	0
Sup F	3	2
Varol HA	3	1
Zhang M	3	1

University of Washington, Northwestern University, and Vanderbilt University contributed the most to the top cited articles with nine, eight, and five articles, respectively. Karolinska Institute and Rehabilitation Institute of Chicago had the next highest rates of top cited articles. Several articles were the result of national or international collaborations between institutions. Table 4 shows the top 10 institutions that contributed to the top cited articles.

**Table 4:** Top ten institutions contributing to the most cited articles in prosthetics

**Institution**	**No. of articles**
University of Washington	9
Northwestern University	8
Vanderbilt University	5
Karolinska Institutet	4
Rehabilitation Institute of Chicago	4
Malmo University Hospital	3
Prosthetics Research Study, Seattle	3
Scuola Superiore Sant’ Anna	3
University of Southampton	3
University of Toronto	3

**Figure 5:**
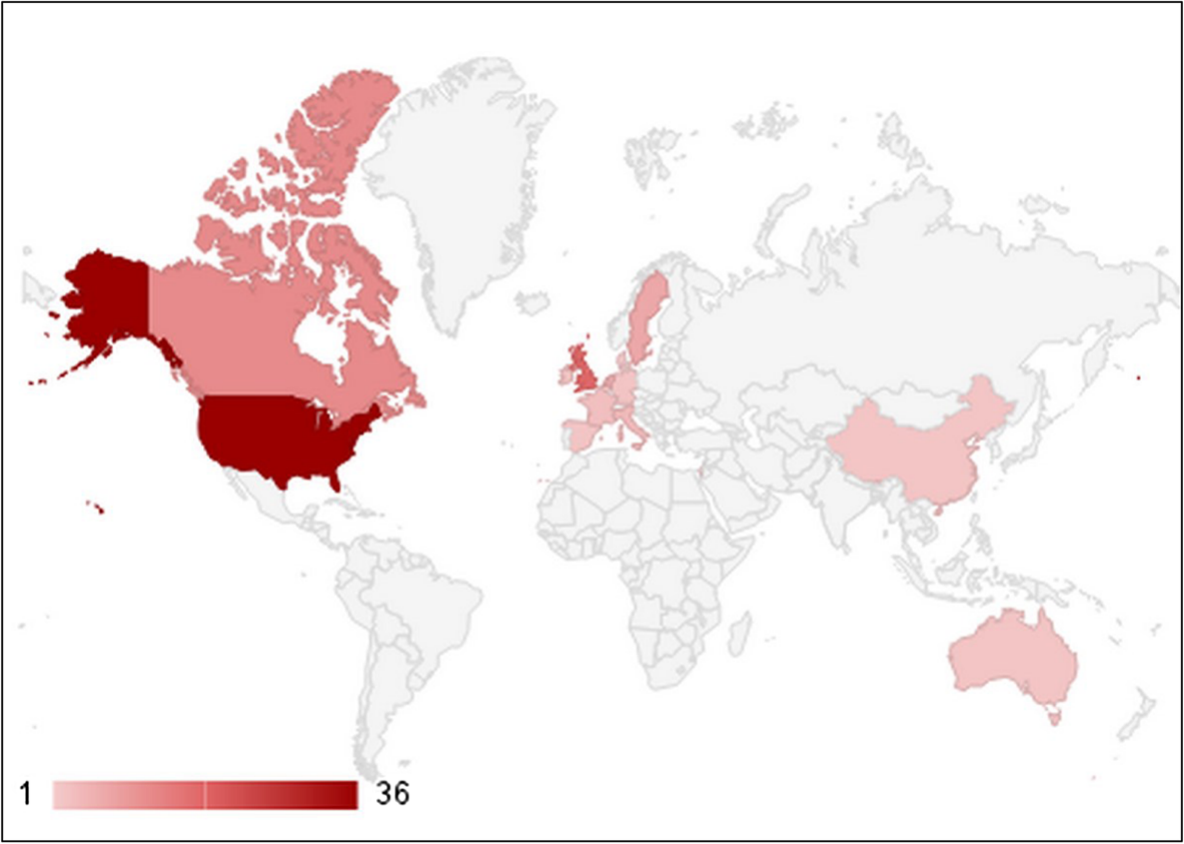
The percentage of top cited articles in different countries.

The origins of the articles were the United States (n = 36), United Kingdom (n = 18), Canada (n = 12), Sweden (n = 7), the Netherlands (n = 6), and Italy (n = 4). The rest of the countries had less than three publications. Figure 5 shows the distribution of top cited articles over the countries of origin. The articles from the United States obtained total of 901 citations, whereas those from the United Kingdom and Canada had 527 and 279 citations, respectively.

## Discussion

The field of prosthetics and orthotics used to be considered an empirical field. However, the knowledge base that developed during the last two decades is being used to advance the field as a science. Generally, in every discipline, a number of studies have a significant impact on the development of a given discipline because they provide the basis for new procedures, methods, or concepts. Articles with the highest impact were obtained by counting the citation (the number of citation times for article) [7]. Through a systematic citation analysis, the current study aimed to identify scientific works that have motivated the practice of prosthetics.

Although prostheses are mainly used to rehabilitate those with amputation, articles related to prostheses can be found in categories other than rehabilitation, including engineering, materials, neurosciences, or sport sciences.

The journal impact factor is one of the known measures that measures a journal’s significance within the corresponding field [13]. In the current study, the journals were obtained from various fields, and majority of which were from the field of rehabilitation, followed by orthopedics, sport sciences, and biomedical engineering. Majority of the top cited papers were published in journals with high impact factors (Table 5). Considering the Web of Science categories, majority of the papers related to prosthesis were published in the journals of Rehabilitation and Orthopedics compared with the field of Engineering. However, this result could be misleading because several journals are categorized under two or more categories.

**Table 5:** Top 10 journals, number of top cited articles and impact factors

**Journal title**	**No. of papers**	**Impact factor**	
		**2010**	**2011**
J Rehabil Res Dev	16	1.708	1.779
Arch Phys Med Rehabil	11	2.254	2.284
Prosthet Orthot Int	9	0.634	0.950
Gait posture	8	2.313	2.123
IEEE Tran Biomed Eng	6	1.790	2.278
Disabil Rehabil	5	1.489	1.498
Clin Biomech	4	2.036	2.071
Med Eng Phys	4	1.909	1.823
Am J Phys Med	3	1.762	1.581
Annal Biomed Eng	3	2.376	2.368

Although we were expecting a higher number, only 4% of the papers were review articles or meta-analysis, whereas majority included original research articles. These results are in contrast with the general belief that review articles had the highest number of citations [14,15]. Our findings are consistent with those of previous studies on top cited articles in other fields [4,16,17]. The findings might be associated with a preference for referring to experimental evidence rather than review articles or expert opinion.

Socket configuration is considered a key element in the field of limb prosthetics because the hard or soft (liner) socket is the immediate interface between the artificial limb and body. Ineffective or uncomfortable socket undermines the function of other components even if they are highly advanced [18]. Three reviews addressed the socket, and this number corresponds to the importance of research on sockets.

The ten-year span of 2001 to 2011 had the highest number of top cited articles. The least number of articles were published in the 1980s. The citation rate has gradually risen over the last decade and is indicative of an increasing consideration for the field of prosthetics. This increase would seem logical because scientific papers are usually cited one or two years after publication and reach peak citation about 10 years after publication [19]. The chronological trend of top cited articles is in accordance with previous findings, and the results indicate that the peak recognition of important papers in a field can be obtained in a 10- to 20-year period [20].

Only 4% of the top cited articles came from Asia, and the majority originated from the United States. Scandinavian countries contributed to the list with eight papers. This result is in line with previous studies [4] and is in accordance with the reported strong influence of the United States in research related to health science [3,17,21-23]. The dominance of the United States can be attributed to high research funding and a large community of American scientists. Moreover, authors from the United States usually prefer to publish in American journals and are more likely to cite other American papers [24]. Most Asian countries have been experiencing war, natural disasters, and landmine issues that have led to many amputations in the last 20 years. Thus, these events intensified the need to conduct studies to address prosthetic concerns. No relationship between country and citation (*P* = .751) was observed, which indicates that a study does not necessarily have to originate from a particular country for it to become top cited.

The subject of articles published in a journal mirrors both the tendency of the editorial board and interest of the authors. However, the citation rate of an article represents its influence on the readers. Therefore, papers addressing the lower limb were more relevant than papers addressing the upper limb. From the articles on the lower limb, the TT level was the most common amputation level of interest, whereas knee disarticulation and Syme’s were the least studied. Moreover, no top cited article was found on partial foot amputation. In the upper limb, the TR amputation level attracted the highest attention from scholars, whereas partial limb amputation was the least studied topic. These findings for both upper and lower limbs might be explained either by the higher rate of particular amputation levels over others or the limitation in recruiting subjects willing to participate in a study.

We likewise tested the hypothesis that several countries might have worked more on upper or lower limb prosthetics. No significant correlation was found between countries and limb prosthetics (*P* = .254). Moreover, the fact that articles could obtain a higher citation if they were concentrated on upper or lower limb prosthetics could not be proven (*P* > .05).

Only 27% of the studies on upper limb addressed more than one amputation level, which indicates that researchers mainly preferred to concentrate on one amputation level. Similarly, most of the lower limb articles tended to focus only on one amputation level.

From the original research papers, 60% had a quantitative research design, whereas 39% were qualitative. This finding might indicate the need for more qualitative studies, primarily on the satisfaction and quality of life of end users with prosthetic services. A prosthetic would not be of benefit if a user is not satisfied, regardless of how advanced the component is. Therefore, more qualitative studies are necessary to differentiate between weakly fitted or designed prosthetic component because it could help increase an individual’s willingness to accept prosthetic services, especially on upper limb prostheses. A stronger communication between professionals and consumers may likewise lead to higher rehabilitation success rates.

Majority of the studies were classified as providing lower levels of evidence, and only a small number of studies were classified as level 1 (RCT). This finding might be attributed to the relative ease in performing simpler designs of study in rehabilitation. No correlation between the level of evidence and number of citations were observed in our study (*P* = .404) despite the common belief that RCT studies are essentially cited more often than the other study designs.

### Study limitations

We acknowledge that our study has several possible methodological limitations. First, the journals have different approaches to accept or reject a submitted manuscript. Thus, particular journals could have stricter selection criteria that might have affected the clinical applicability or quality of their publications. The criteria could be a reason why most top cited papers were found in one journal. Second, challenges and problems might arise from citation counts, such as ignoring potential citations in book chapters, considering self-citations, peers’ preference to cite papers from the journal they submit their work, and preference to cite review articles or full-length articles. Quality of a work is best recognized by citation count and is a superior measure of an author’s impact and originality of work compared with article counts [25]. Third, the number of citations and citation index of a particular paper could be influenced by its publication year because some journals check the quality of a submitted work based on the usage of recently published papers. We did not find any preference for more recent articles. Next, chronological bias is potential in the current citation analysis because older articles can still be cited more frequently regardless of actual impact, whereas articles published more recently had insufficient time to have high citation rates. Consequently, recently published articles might have been underestimated in terms of their impact [25]. Thus, the likelihood of biases in citing a given article as a result of tendency to cite papers by colleagues, well-known authors, or reviewers should be acknowledged. The influence of self-citations should also be taken into account [26].

## Conclusions

This study might be considered as the first report on the most cited papers in prosthetics. The findings indicate that studies written in English concentrated mainly on lower limb prostheses and published in high-impact rehabilitation journals are the most likely papers to be cited in the prosthetics field. Additionally, transtibial and transradial limb prosthetics were more frequently discussed in top cited articles. The articles were mainly experimental designs. The findings imply that the rate of citation does not necessarily determine the importance or impact of a specific publication.

## Abbreviations

KD: Knee disarticulation; RCT: Randomized controlled trial; SD: Shoulder disarticulation; TF: Transfemoral; TH: Transhumeral; TR: Transradial; TT: Transtibial.

## Competing interests

The authors declare no competing interests.

## Authors’ contributions

AE and HG carried out the research and drafted the manuscript. SA and NAAO and BS participated in its design, performed the statistical analysis and revised it critically for important intellectual content. All authors read and approved the final manuscript.

## Supplementary Material

Additional file 1: Table S1. The 100 top cited articles in the field of limb prosthetics. Click here for file
