# Supplementary material for: 100 top-cited scientific papers in limb prosthetics
Source: Biomed Eng Online. 2013 Nov 17;12:119. doi: 10.1186/1475-925X-12-119 (PMC3842667; doi:10.1186/1475-925X-12-119)
Supplement: Additional file 1: Table S1. — The 100 top cited articles in the field of limb prosthetics. [file 1475-925X-12-119-S1.doc]

Table 1: The 100 top cited articles in the field of limb prosthetics.

| **Article** | **No. of Citations** | **Citation Index** |
| --- | --- | --- |
| Kuiken T, Dumanian G, Lipschutz R, Miller L, Stubblefield K. The use of targeted muscle reinnervation for improved myoelectric prosthesis control in a bilateral shoulder disarticulation amputee. Prosthet Orthot Int. 2004;28:245-53. | 90 | 11.3 |
| Houghton A, Taylor P, Thurlow S, Rootes E, McColl I. Success rates for rehabilitation of vascular amputees: implications for preoperative assessment and amputation level. Brit J Surg. 1992;79:753-5. | 89 | 4.5 |
| Jacobson SC, Knutti DF, Johnson RT, Sears HH. Development of the Utah artificial arm. IEEE Trans Biomed Eng. 1982; 29:249-69. | 83 | 2.8 |
| Schmalz T, Blumentritt S, Jarasch R. Energy expenditure and biomechanical characteristics of lower limb amputee gait: The influence of prosthetic alignment and different prosthetic components. Gait posture. 2002;16:255-63. | 71 | 7.1 |
| McWhinnie D, Gordon A, Collin J, Gray D, Morrison J. Rehabilitation outcome 5 years after 100 lower‐limb amputations. Brit J Surg. 1994;81:1596-9. | 67 | 3.7 |
| Karlik B, Osman Tokhi M, Alci M. A fuzzy clustering neural network architecture for multifunction upper-limb prosthesis. IEEE Trans Biomed Eng. 2003;50:1255-61. | 63 | 7.0 |
| Light C, Chappell P. Development of a lightweight and adaptable multiple-axis hand prosthesis. Med Eng Phys. 2000;22:679-84. | 53 | 4.4 |
| Legro MW, Reiber G, del Aguila M, Ajax MJ, Boone DA, Larsen JA, et al. Issues of importance reported by persons with lower limb amputations and prostheses. J Rehab Res Dev. 1999;36:155-63. | 51 | 3.9 |
| Nolan L, Wit A, Dudziñski K, Lees A, Lake M, Wychowañski M. Adjustments in gait symmetry with walking speed in trans-femoral and trans-tibial amputees. Gait Posture. 2003;17:142-51. | 45 | 5.0 |
| Sanderson DJ, Martin PE. Lower extremity kinematic and kinetic adaptations in unilateral below-knee amputees during walking. Gait Posture. 1997;6:126-36. | 43 | 2.9 |
| Isakov E, Mizrahi J, Ring H, Susak Z, Hakim N. Standing sway and weight-bearing distribution in people with below-knee amputations. Arch Phys Med Rehabil. 1992;73:174. | 43 | 2.2 |
| Pons J, Rocon E, Ceres R, Reynaerts D, Saro B, Levin S, et al. The MANUS-HAND dextrous robotics upper limb prosthesis: mechanical and manipulation aspects. Autonom Robot. 2004;16:143-63. | 40 | 5.0 |
| Hof AL, van Bockel RM, Schoppen T, Postema K. Control of lateral balance in walking: experimental findings in normal subjects and above-knee amputees. Gait Posture. 2007;25:250-8. | 39 | 7.8 |
| Pezzin LE, Dillingham TR, MacKenzie EJ, Ephraim P, Rossbach P. Use and satisfaction with prosthetic limb devices and related services. Arch Phys Med Rehabil. 2004;85:723-9. | 39 | 4.9 |
| Carrozza M, Massa B, Micera S, Lazzarini R, Zecca M, Dario P. The development of a novel prosthetic hand-ongoing research and preliminary results. IEEE/ASME Tran Mech. 2002;7:108-14. | 37 | 3.7 |
| Sup F, Bohara A, Goldfarb M. Design and control of a powered transfemoral prosthesis. Int J Robot Res. 2008;27:263-73. | 36 | 9.0 |
| Farrell TR, Weir RF. The optimal controller delay for myoelectric prostheses. IEEE Tran Neur Sys Rehabil Eng. 2007;15:111-8. | 36 | 7.2 |
| Holden JM, Fernie GR. Extent of artificial limb use following rehabilitation. J Orthop Res. 1987;5:562-8. | 36 | 1.4 |
| Ehrsson HH, Rosén B, Stockselius A, Ragnö C, Köhler P, Lundborg G. Upper limb amputees can be induced to experience a rubber hand as their own. Brain. 2008;131:3443-52. | 35 | 8.8 |
| Segal AD, Orendurff MS, Klute GK, McDowell ML, Pecoraro JA, Shofer J, et al. Kinematic and kinetic comparisons of transfemoral amputee gait using C-Leg® and Mauch SNS® prosthetic knees. J Rehab Res Dev. 2006;43:857. | 35 | 5.8 |
| Seroussi RE, Gitter A, Czerniecki JM, Weaver K. Mechanical work adaptations of above-knee amputee ambulation. Arch Phys Med Rehabil. 1996;77:1209-14. | 35 | 2.2 |
| Franchignoni F, Orlandini D, Ferriero G, Moscato TA. Reliability, validity, and responsiveness of the locomotor capabilities index in adults with lower-limb amputation undergoing prosthetic training. Arch Phys Med Rehabil. 2004;85:743-8. | 34 | 4.3 |
| Levy S. Amputees: skin problems and prostheses. Cutis; cutaneous medicine for the practitioner. 1995;55:297-301. | 32 | 1.9 |
| Cardinal BJ, Kosma M, McCubbin JA. Factors influencing the exercise behavior of adults with physical disabilities. Med Sci Sport Exerc. 2004;36:868-75. | 31 | 3.9 |
| Lyon CC, Kulkarni J, Zimersonc E, Van Ross E, Beck MH. Skin disorders in amputees. J Am Acad Dermatol. 2000;42:501-7. | 30 | 2.5 |
| Biddiss EA, Chau TT. Upper limb prosthesis use and abandonment: A survey of the last 25 years. Prosthet Orthot Int. 2007;31:236-57. | 29 | 5.8 |
| Whyte A, Carroll L. A preliminary examination of the relationship between employment, pain and disability in an amputee population. Disabil Rehabil. 2002;24:462-70. | 28 | 2.8 |
| Sanders JE, Daly CH, Burgess EM. Interface shear stresses during arnbulation with a below-knee prosthetic limb. J Rehabil Res Dev. 1992;29:1-8. | 28 | 1.4 |
| Sebelius FC, Rosen BN, Lundborg GN. Refined myoelectric control in below-elbow amputees using artificial neural networks and a data glove. J Hand Surg. 2005;30:780-9. | 27 | 3.9 |
| Murray C, Fox J. Body image and prosthesis satisfaction in the lower limb amputee. Disabil Rehabil. 2002;24:925-31. | 27 | 2.7 |
| Pinzur MS, Angelats J, Light TR, Izuierdo R, Pluth T. Functional outcome following traumatic upper limb amputation and prosthetic limb fitting. J Hand Surg. 1994;19:836-9. | 26 | 1.4 |
| Engsberg JR, Lee AG, Patterson JL, Harder JA. External loading comparisons between able-bodied and below-knee-amputee children during walking. Arch Phys Med Rehabil. 1991;72:657-61. | 24 | 1.1 |
| Miller LA, Stubblefield KA, Lipschutz RD, Lock BA, Kuiken TA. Improved myoelectric prosthesis control using targeted reinnervation surgery: a case series. IEEE Tran Neur Sys Rehabil Eng. 2008;16:46-50. | 23 | 5.8 |
| Gallagher P, MacLachlan M. The Trinity Amputation and Prosthesis Experience Scales and quality of life in people with lower-limb amputation. Arch Phys Med Rehabil. 2004;85:730-6. | 23 | 2.9 |
| Miller W, Deathe A. A prospective study examining balance confidence among individuals with lower limb amputation. Disabil Rehabil. 2004;26:875-81. | 22 | 2.8 |
| English C, Russell D. Implementation of variable joint stiffness through antagonistic actuation using rolamite springs. Mech Mach Theory. 1999;34:27-40. | 22 | 1.7 |
| Zhang M, Turner-Smith A, Roberts V, Tanner A. Frictional action at lower limb/prosthetic socket interface. Med Eng Phys. 1996;18:207-14. | 22 | 1.4 |
| Bilodeau S, Hébert R, Desrosiers J. Lower limb prosthesis utilisation by elderly amputees. Prosthet Orthot Int. 2000;24:126-32. | 21 | 1.8 |
| Vinet R, Lozac'h Y, Beaudry N, Drouin G. Design methodology for a multifunctional hand prosthesis. J Rehab Res Dev. 1995;32:316-324. | 21 | 1.2 |
| Yang J, Pitarch EP, Abdel-Malek K, Patrick A, Lindkvist L. A multi-fingered hand prosthesis. Mech Mach Theory. 2004;39:555-81. | 20 | 2.5 |
| Lundborg G, Rosen B. Sensory substitution in prosthetics. Hand Clin. 2001;17:481-8. | 20 | 1.8 |
| Gitter A, Czerniecki J, Meinders M. Effect of Prosthetic Mass on Swing Phase Work During Above-Knee Amputee Ambulation1. Am J Phys Med Rehabil. 1997;76:114-21. | 20 | 1.3 |
| Czerniecki JM, Gitter A. Insights into amputee running: a muscle work analysis. Am J Phys Med Rehabil. 1992;71:209-18. | 19 | 1.0 |
| Chappell P, Kyberd PJ. Prehensile control of a hand prosthesis by a microcontroller. J Biomed Eng. 1991;13:363-9. | 19 | 0.9 |
| Biddiss E, Chau T. Upper-limb prosthetics: critical factors in device abandonment. Am J Phys Med Rehabil. 2007;86:977-87. | 18 | 3.6 |
| Pylatiuk C, Schulz S, Döderlein L. Results of an Internet survey of myoelectric prosthetic hand users. Prosthet Orthot Int. 2007;31:362-70. | 18 | 3.6 |
| Lee WC, Frossard LA, Hagberg K, Haggstrom E, Brånemark R, Evans JH, et al. Kinetics of transfemoral amputees with osseointegrated fixation performing common activities of daily living. Clin Biomech. 2007;22:665-73. | 18 | 3.6 |
| Jia X, Koenig MA, Zhang X, Zhang J, Chen T, Chen Z. Residual motor signal in long-term human severed peripheral nerves and feasibility of neural signal-controlled artificial limb. J Hand Surg. 2007;32:657-66. | 18 | 3.6 |
| Buckley JG. Biomechanical adaptations of transtibial amputee sprinting in athletes using dedicated prostheses. Clin Biomech. 2000;15:352-8. | 18 | 1.5 |
| de Visser H, Herder JL. Force-directed design of a voluntary closing hand prosthesis. J Rehab Res Dev. 2000;37:261-72. | 18 | 1.5 |
| Michaud SB, Gard SA, Childress DS. A preliminary investigation of pelvic obliquity patterns during gait in persons with transtibial and transfemoral amputation. J Rehab Res Dev. 2000;37:1-10. | 18 | 1.5 |
| Dalley SA, Wiste TE, Withrow TJ, Goldfarb M. Design of a multifunctional anthropomorphic prosthetic hand with extrinsic actuation. IEEE/ASME Tran Mech. 2009;14:699-706. | 17 | 5.7 |
| Fergason J, Smith DG. Socket considerations for the patient with a transtibial amputation. Clin Orthop Rel Res. 1999;361:76-84. | 17 | 1.3 |
| Rossi SA, Doyle W, Skinner HB. Gait initiation of persons with below-knee amputation: The characterization and comparison of force profiles. J Rehab Res Dev. 1995;32:120-7. | 17 | 1.0 |
| Marasco PD, Schultz AE, Kuiken TA. Sensory capacity of reinnervated skin after redirection of amputated upper limb nerves to the chest. Brain. 2009;132:1441-8. | 16 | 5.3 |
| Vanicek N, Strike S, McNaughton L, Polman R. Gait patterns in transtibial amputee fallers vs. non-fallers: Biomechanical differences during level walking. Gait Posture. 2009;29:415-20. | 16 | 5.3 |
| Lee WC, Zhang M. Design of monolimb using finite element modelling and statistics-based Taguchi method. Clin Biomech. 2005;20:759-66. | 16 | 2.3 |
| Hagberg K, Branemark R, Hagg O. Questionnaire for Persons with a Transfemoral Amputation (Q-TFA): initial validity and reliability of a new outcome measure. J Rehab Res Dev. 2004;41:695-706. | 16 | 2.0 |
| Van Damme H, Rorive M, Martens De Noorthout B, Quaniers J, Scheen A, Limet R. Amputations in diabetic patients: a plea for footsparing surgery. Acta Chirug Belg. 2001; 101:123-9. | 16 | 1.5 |
| Jensen JS, Heim S. Evaluation of polypropylene prostheses designed by the International Committee of the Red Cross for trans-tibial amputees. Prosthet Orthot Int. 2000;24:47-54. | 16 | 1.3 |
| English RD, Hubbard W, McElroy G. Establishment of consistent gait after fitting of new components. J Rehab Res Dev. 1995; 32:32-35. | 16 | 0.9 |
| Meek S, Fetherston S. Comparison of signal-to-noise ratio of myoelectric filters for prosthesis control. J Rehab Res Dev. 1992;29:9-20. | 16 | 0.8 |
| Mann RW. Cybernetic limb prosthesis: The ALZA distinguished lecture. Annal Biomed Eng. 1981;9:1-43. | 16 | 0.5 |
| Varol HA, Sup F, Goldfarb M. Multiclass real-time intent recognition of a powered lower limb prosthesis. IEEE Trans Biomed Eng. 2010;57:542-51. | 15 | 7.5 |
| Stineman MG, Kwong PL, Kurichi JE, Prvu-Bettger JA, Vogel WB, Maislin G, et al. The effectiveness of inpatient rehabilitation in the acute postoperative phase of care after transtibial or transfemoral amputation: study of an integrated health care delivery system. Arch Phys Med Rehabil. 2008;89:1863-72. | 15 | 3.8 |
| Vrieling A, Van Keeken H, Schoppen T, Otten E, Halbertsma J, Hof A, et al. Gait initiation in lower limb amputees. Gait Posture. 2008;27:423-30. | 15 | 3.8 |
| Dudek NL, Marks MB, Marshall SC, Chardon JP. Dermatologic conditions associated with use of a lower-extremity prosthesis. Arch Phys Med Rehabil. 2005;86:659-63. | 15 | 2.1 |
| van der Linde H, Hofstad CJ, Geurts AC, Postema K, Geertzen JH, Van Limbeek J. A systematic literature review of the effect of different prosthetic components on human functioning with a lower-limb prosthesis. J Rehab Res Dev. 2004;41:555-70. | 15 | 1.9 |
| Legro MW, Reiber GE, Czerniecki JM, Sangeorzan BJ. Recreational activities of lower-limb amputees with prostheses. J Rehab Res Dev. 2001;38:319-26. | 15 | 1.4 |
| Viton J, Mouchnino L, Mille M, Cincera M, Delarque A, Pedotti A, et al. Equilibrium and movement control strategies in trans-tibial amputees. Prosthet Orthot Int. 2000;24:108-16. | 15 | 1.3 |
| Tufa A, Lamberti C, Davalli A, Sacchetti R. Experimental development of a sensory control system for an upper limb myoelectric prosthesis with cosmetic covering. J Rehab Res Dev. 1998;35:14-26. | 15 | 1.1 |
| He P, Xue K, Murka P. 3-D imaging of residual limbs using ultrasound. J Rehab Res Dev. 1997;34:269-78. | 15 | 1.0 |
| Popović D, Oĝuztöreli M, Stein R. Optimal control for the active above-knee prosthesis. Annal Biomed Eng. 1991;19:131-50. | 15 | 0.7 |
| Sup F, Varol HA, Mitchell J, Withrow TJ, Goldfarb M. Preliminary evaluations of a self-contained anthropomorphic transfemoral prosthesis. IEEE/ASME Tran Mech. 2009;14:667-76. | 14 | 4.7 |
| Engeberg ED, Meek SG, Minor MA. Hybrid force–velocity sliding mode control of a prosthetic hand. IEEE Trans Biomed Eng. 2008;55:1572-81. | 14 | 3.5 |
| Portnoy S, Yarnitzky G, Yizhar Z, Kristal A, Oppenheim U, Siev-Ner I, et al. Real-time patient-specific finite element analysis of internal stresses in the soft tissues of a residual limb: a new tool for prosthetic fitting. Annal Biomed Eng. 2007;35:120-35. | 14 | 2.8 |
| Kenney L, Lisitsa I, Bowker P, Heath G, Howard D. Dimensional change in muscle as a control signal for powered upper limb prostheses: a pilot study. Med Eng Phys. 1999;21:589-97. | 14 | 1.1 |
| Taha Z, Brown R, Wright D. Modelling and simulation of the hand grasping using neural networks. Med Eng Phys. 1997;19:536-8. | 14 | 0.9 |
| Marasco PD, Kim K, Colgate JE, Peshkin MA, Kuiken TA. Robotic touch shifts perception of embodiment to a prosthesis in targeted reinnervation amputees. Brain. 2011;134:747-58. | 13 | 13.0 |
| Rosén B, Ehrsson HH, Antfolk C, Cipriani C, Sebelius F, Lundborg G. Referral of sensation to an advanced humanoid robotic hand prosthesis. Scand J Plast Reconstr Surg Hand Surg. 2009;43:260-6. | 13 | 4.3 |
| Hermansson L, Fisher A, Bernspang B, Eliasson A-C. Assessment of capacity for myoelectric control: a new Rasch-built measure of prosthetic hand control. J Rehabil Med. 2005;37:166-71. | 13 | 1.9 |
| Pillet J, Didierjean-Pillet A. Aesthetic hand prosthesis: gadget or therapy? Presentation of a new classification. J Hand Surg (British and European Volume). 2001;26:523-8. | 13 | 1.2 |
| Wong CK, Edelstein JE. Unna and elastic postoperative dressings: Comparison of their effects on function of adults with amputation and vascular disease. Arch Phys Med Rehabil. 2000;81:1191-8. | 13 | 1.1 |
| Hill SW, Patla AE, Ishac MG, Adkin AL, Supan TJ, Barth DG. Kinematic patterns of participants with a below-knee prosthesis stepping over obstacles of various heights during locomotion. Gait Posture. 1997;6:186-92. | 13 | 0.9 |
| Miller LA, Childress DS. Analysis of a vertical compliance prosthetic foot. J Rehab Res Dev. 1997;34:52-7. | 13 | 0.9 |
| Esquenazi A, Meier RH. Rehabilitation in limb deficiency. 4. Limb amputation. Arch Phys Med Rehabil. 1996;77:S18-S28. | 13 | 0.8 |
| Kyberd P, Mustapha N, Carnegie F, Chappell P. A clinical experience with a hierarchically controlled myoelectric hand prosthesis with vibro-tactile feedback. Prosthet Orthot Int. 1993;17:56-64. | 13 | 0.7 |
| Cipriani C, Controzzi M, Carrozza MC. Objectives, criteria and methods for the design of the SmartHand transradial prosthesis. Robotica. 2010;28:919-27. | 12 | 6.0 |
| Beyaert C, Grumillier C, Martinet N, Paysant J, André J-M. Compensatory mechanism involving the knee joint of the intact limb during gait in unilateral below-knee amputees. Gait Posture. 2008;28:278-84. | 12 | 3.0 |
| Atherton R, Robertson N. Psychological adjustment to lower limb amputation amongst prosthesis users. Disabil Rehabil. 2006;28:1201-9. | 12 | 2.0 |
| Lowery MM, Weir RF, Kuiken TA. Simulation of intramuscular EMG signals detected using implantable myoelectric sensors (IMES). IEEE Trans Biomed Eng. 2006;53:1926-33. | 12 | 2.0 |
| Pons J, Ceres R, Rocon E, Levin S, Markovitz I, Saro B, et al. Virtual reality training and EMG control of the MANUS hand prosthesis. Robotica. 2005;23:311-7. | 12 | 1.7 |
| Baars E, Geertzen J. Literature review of the possible advantages of silicon liner socket use in trans-tibial prostheses. Prosthet Orthot Int. 2005;29:27-37. | 12 | 1.7 |
| Van der Linden M, Twiste N, Rithalia S. The biomechanical effects of the inclusion of a torque absorber on trans-femoral amputee gait, a pilot study. Prosthet Orthot Int. 2002;26:35-43. | 12 | 1.2 |
| Lundborg G, Rosen B, Lindström K, Lindberg S. Artificial sensibility based base on the use of piezoresistive sensors: Preliminary observations. J Hand Surg: British & European Volume. 1998;23:620-6. | 12 | 0.9 |
| Ha KH, Varol HA, Goldfarb M. Volitional control of a prosthetic knee using surface electromyography. IEEE Trans Biomed Eng. 2011;58:144-51. | 11 | 11.0 |
| Carey SL, Jason Highsmith M, Maitland ME, Dubey RV. Compensatory movements of transradial prosthesis users during common tasks. Clin Biomech. 2008;23:1128-35. | 11 | 2.8 |
| Saradjian A, Thompson AR, Datta D. The experience of men using an upper limb prosthesis following amputation: positive coping and minimizing feeling different. Disabil Rehabil. 2008;30:871-83. | 11 | 2.8 |
| Lee WC, Zhang M, Boone D, Contoyannis B. Finite-element analysis to determine effect of monolimb flexibility on structural strength and interaction between residual limb and prosthetic socket. J Rehab Res Dev. 2004;41:775-86. | 11 | 1.4 |
| Buckley JG. Sprint kinematics of athletes with lower-limb amputations. Arch Phys Med Rehabil. 1999;80:501-8. | 11 | 0.8 |
